# Glutathione ethyl ester reverses the deleterious effects of fentanyl on ventilation and arterial blood-gas chemistry while prolonging fentanyl-induced analgesia

**DOI:** 10.1038/s41598-021-86458-x

**Published:** 2021-03-26

**Authors:** Michael W. Jenkins, Faiza Khalid, Santhosh M. Baby, Walter J. May, Alex P. Young, James N. Bates, Feixiong Cheng, James M. Seckler, Stephen J. Lewis

**Affiliations:** 1 Department of Biomedical Engineering, Case Western Reserve University, Cleveland, OH USA; 2 Department of Pediatrics, Case Western Reserve University, 10900 Euclid Avenue, Cleveland, OH 44106-4984 USA; 3 Department of Internal Medicine, University Hospitals, Case Western Reserve University, Cleveland, OH USA; 4 Section of Biology, Galleon Pharmaceuticals, Inc, Horsham, PA USA; 5 Department of Pediatrics, University of Virginia, Charlottesville, VA USA; 6 Department of Anesthesia, University of Iowa, Iowa City, Iowa, USA; 7 Cleveland Clinic Lerner College of Medicine, Case Western Reserve University, Cleveland, OH USA; 8 Department of Pharmacology, Case Western Reserve University, Cleveland, OH USA; 9 Present Address: Translational Sciences Treatment Discovery, Galvani Bioelectronics, Inc, 1250 S Collegeville Rd, Collegeville, PA 1r9426 USA

**Keywords:** Drug discovery, Systems biology

## Abstract

There is an urgent need to develop novel compounds that prevent the deleterious effects of opioids such as fentanyl on minute ventilation while, if possible, preserving the analgesic actions of the opioids. We report that L-glutathione ethyl ester (GSHee) may be such a novel compound. In this study, we measured tail flick latency (TFL), arterial blood gas (ABG) chemistry, Alveolar-arterial gradient, and ventilatory parameters by whole body plethysmography to determine the responses elicited by bolus injections of fentanyl (75 μg/kg, IV) in male adult Sprague–Dawley rats that had received a bolus injection of GSHee (100 μmol/kg, IV) 15 min previously. GSHee given alone had minimal effects on TFL, ABG chemistry and A-a gradient whereas it elicited changes in some ventilatory parameters such as an increase in breathing frequency. In vehicle-treated rats, fentanyl elicited (1) an increase in TFL, (2) decreases in pH, pO_2_ and sO_2_ and increases in pCO_2_ (all indicative of ventilatory depression), (3) an increase in Alveolar-arterial gradient (indicative of a mismatch in ventilation-perfusion in the lungs), and (4) changes in ventilatory parameters such as a reduction in tidal volume, that were indicative of pronounced ventilatory depression. In GSHee-pretreated rats, fentanyl elicited a more prolonged analgesia, relatively minor changes in ABG chemistry and Alveolar-arterial gradient, and a substantially milder depression of ventilation. GSHee may represent an effective member of a novel class of thiolester drugs that are able to prevent the ventilatory depressant effects elicited by powerful opioids such as fentanyl and their deleterious effects on gas-exchange in the lungs without compromising opioid analgesia.

## Introduction

Fentanyl is a high-potency opioid receptor (OR) agonist prescribed to treat pain^1,2^. The misuse-abuse of fentanyl causes adverse effects including opioid-induced respiratory depression (OIRD)^1^. Fentanyl has high affinity for μ-ORs^3–6^ and also activates δ- and κ-ORs^7–10^. Mechanisms underlying the effects of fentanyl have been studied^11–14^. Henderson et al.^15^ found that fentanyl-induced analgesia, decreased tidal volume (Vt) and increased Alveolar-arterial (A-a) gradient (ventilation-perfusion mismatch)^16^, was reduced by a peripherally-restricted μ-OR antagonist, naloxone methiodide. This agrees with reports that the analgesia and OIRD elicited by morphine and heroin are attenuated by naloxone methiodide^17–19^. As such, fentanyl may act on peripheral structures, brain regions without a blood–brain barrier, and brain structures within the blood brain barrier^15,20^.

Intravenous L-cysteine ethyl ester (L-CYSee) reverses the effects of morphine on arterial blood-gas (ABG) chemistry in tracheotomized rats^21^. L-CYSee is membrane-permeable^22,23^, readily enters peripheral tissues and brain^24^, and increases intracellular pools of cysteine^25,26^ via a membrane-associated carboxylesterase^27^. Increased availability of cysteine alters redox status of cells^28,29^ and enhances production of glutathione (GSH)^30,31^, which exerts redox effects and S-glutathiolation of proteins^32^, and production of hydrogen sulfide^30,31^, which increases minute ventilation (Ve) via actions in carotid bodies^33^. Enhanced biovailability of L-cysteine and L-GSH promotes formation of S-nitrosothiols such as S-nitrosocysteine and S-nitrosylation status of functional proteins^34–37^. S-nitrosothiols exert diverse effects by the S-nitrosylation of functional proteins^38,39^ and S-nitrosothiols in brainstem^40^ and peripheral structures^41–43^ exert positive effects on ventilatory function. In contrast, there is evidence that morphine alters redox status of cells to an oxidative state^44^ and reduces cellular GSH levels^45^.

Mendoza et al.^21^ reasoned that L-CYSee or its free radical cation^46^ reversed the effects of morphine via its reductive capacity, which down-regulates ORs^47^ or affects OR function by effects on membrane-associated proteins^48^. The efficacy of L-CYSee raises the question as to whether L-GSH ethyl ester (GSHee) can modulate OIRD. GSHee is hydrolyzed to GSH by esterases in plasma and cells^49–51^. Peripheral administration of GSHee increases the concentrations of GSHee, GSH and cysteine in blood cells, peripheral organs^49–51^ and cerebrospinal fluid^52^, but not brain tissue^53^. Nonetheless, central administration or direct application of GSHee to isolated neurons elicits robust increases in GSH levels^53^. Elevation of GSH levels by GSHee protects neurons from oxidative or metabolic stress, excitotoxicity, ischemia and ischemia–reperfusion injury^53–64^. GSHee protects endothelial cells^65^ and hepatic mitochondria^66,67^ from endotoxin-induced injury, and protects hepatic mitochondria^68^ and cardiac cells from ischemia–reperfusion injury^69^ and improves their functional recovery. GSHee enhances neuron survival, and stabilizes spinal cord blood flow after injury^70,71^. GSHee also enhances insulin sensitivity^72^, and decreases allergen-induced airway hyper-responsiveness^73,74^.

A unique intracellular enzymatic cascade is involved in the bidirectional conversion of L-cysteine to γ-glutamyl-L-cysteine to γ-L-glutamyl-L-cysteinylglycine (GSH)^75–77^. These compounds play vital roles in maintaining redox homeostasis, protecting cells from oxidative damage and the toxicity of xenobiotic electrophiles^75–77^. Our laboratory is exploring the biological activities and particularly the ventilatory actions of the ethyl and methyl ester derivatives of these thiols against OIRD and as mentioned, we have determined that L-CYSee can elicit immediate reversal of the negative effects of morphine on ABG in anesthetized, tracheotomized rats but it is important to note that L-CYSee was not effective in rats without a tracheotomy, obviously suggesting that L-CYSee had negative effects in the upper airway and that better thiolester therapeutics than L-CYSee need to be considered. Despite the wealth of information about the biological activities of GSH and GSHee, the possibility that GSHee has the ability to modulate any of the pharmacological effects of opioids has not been explored to date. On the basis of the evidence presented above, the major objectives of the present study were (1) to determine the effects of GSHee on ventilatory performance in freely-moving adult rats, and (2) to determine whether the prior administration of GSHee is able to ameliorate the negative effects of fentanyl on breathing and gas-exchange in freely-moving adult rats. The results demonstrate that GSHee (100 μmol/kg, IV) has pronounced positive effects on breathing in freely-moving adult male Sprague–Dawley rats and that it markedly attenuates the deleterious effects elicited by subsequent injection of fentanyl (75 μg/kg, IV) on minute ventilation, A-a gradient and ABG chemistry while extending the analgesic actions of the OR agonist. In contrast, the injection of GSH itself (100 μmol/kg, IV), while having positive effects on breathing did not attenuate the negative effects of this dose of fentanyl on breathing parameters.

## Methods

### Rats and surgical procedures

All studies were carried out in accordance with the NIH Guide for the Care and Use of Laboratory Animals (NIH Publication No. 80-23) revised in 1996. In addition, all studies were carried out in compliance with the ARRIVE (Animal Research: Reporting of In Vivo Experiments) guidelines (http://www.nc3rs.org.uk/page.asp?id=1357). The protocols were approved by the Animal Care and Use Committees of the University of Virginia and Case Western Reserve University. Adult male Sprague–Dawley rats (Harlan, Madison, WI, USA) were implanted with jugular vein catheters under 2% isoflurane anesthesia and some rats received femoral arterial catheters^15,78,79^. The rats were allowed at least four days to recover from surgery before use. All arterial catheters were flushed daily with heparin solution (50 units heparin in phosphate-buffered saline at 0.1 M, pH 7.4). All catheters were flushed with phosphate-buffered saline (0.1 M, pH 7.4) approximately four hours before commencement of the experiments. All studies were performed in a quiet laboratory with relative humidity of 50 ± 2% and room temperature of 21.3 ± 0.2 °C.

### Antinociception protocols

Antinociception was determined by a radiant heat tail-flick (TF) assay^15,78,79^. The intensity of the light was adjusted so that baseline TF latencies were approximately 3 s. A cutoff time of 12 s was set to minimize damage to the tail. Baseline TF latencies were established and after 15 min, the rats were injected with vehicle (saline, 1 ml/kg, IV; n = 9 rats; 316 ± 2 g) or GSHee (100 μmol/kg, IV; n = 9; 317 ± 2 g) and TF latencies were then recorded after 5 and 15 min. At that point, all of the rats received an injection of fentanyl (75 μg/kg, IV) and TF latencies were recorded at 15, 30, 60, 120, 180 and 240 min thereafter. The data are shown as actual TF latencies (sec) and as “maximum possible effect” (%MPE) using the formula, %MPE = [(post-injection TF latency − baseline TF latency)/(12 − baseline TF latency)] × 100.

### Protocols for blood gas measurements and determination of Arterial-alveolar gradient

Arterial blood samples (100 μL) were taken 15 min before, 1, 7.5 and 15 min after injection of vehicle (saline, IV; n = 9 rats; 322 ± 2 g) or GSHee (100 μmol/kg, IV; n = 9 rats; 320 ± 2 g) and 1, 5, 10, 15 and 20 min after injection of fentanyl (75 μg/kg, IV). The pH, pCO_2_, pO_2_ and sO_2_ of the samples were measured using a Radiometer blood-gas analyzer (ABL800 FLEX). A-a gradient measures the difference between alveolar and arterial blood O_2_ concentrations^15,16,80,81^. A decrease in PaO_2_, without a change in A-a gradient is caused by hypo-ventilation whereas a decrease in PaO_2_ with an increase in A-a gradient indicates ventilation–perfusion mismatch^15,16,82^. A-a gradient = PAO_2_ − PaO_2_, where PAO_2_ is the partial pressure of alveolar O_2_ and PaO_2_ is pO_2_ in arterial blood. PAO_2_ = [(FiO_2_ × (P_atm_ − P_H2O_) − (PaCO_2_/respiratory quotient)], where FiO_2_ is the fraction of O_2_ in inspired air; P_atm_ is atmospheric pressure; P_H2O_ is the partial pressure of H_2_O in inspired air; PaCO_2_ is pCO_2_ in arterial blood; and respiratory quotient (RQ) is the ratio of CO_2_ eliminated/O_2_ consumed. We took FiO_2_ of room-air to be 21% = 0.21, P_atm_ to be 760 mmHg, and P_H2O_ to be 47 mmHg^16,80,81^. We took the RQ value of our adult male rats to be 0.9^83,84^.

### Body temperature (BT) protocols

Changes in BT impact the size of recorded flow-related variables in plethysmography chambers^85^. Our chambers do not monitor BT, but it is imperative to record BT to understand its influence on GSHee on fentanyl-induced changes in ventilation. Adult male Sprague–Dawley rats were placed in separate open plastic boxes and allowed 60–90 min to acclimatize. BT was recorded as described previously^86^. A thermistor probe, inserted 5–6 cm into the rectum to allow regular recording of BT, was connected to a telethermometer (Yellow Springs Instruments) was taped to the tail. BT was recorded every 5 min during acclimatization to establish baseline values. One group of rats received vehicle (saline, 1 ml/kg, IV; n = 9 rats; 321 ± 2 g) and another received GSHee (100 μmol/kg, IV; n = 9 rats; 318 ± 2 g) and BT was recorded at 5 and 15 min post-injection. Both groups then received an injection of fentanyl (75 μg/kg, IV) and BT was recorded 5, 10, 15, 20, 25 and 30 min later.

### Whole-body plethysmography measurement of ventilatory parameters

Ventilatory parameters were recorded in freely-moving rats by whole body plethysmography (PLY3223; Data Sciences International, St. Paul, MN) as described previously^15,87–97^. The rats were allowed 60 min to acclimatize to the chambers and to allow true resting ventilatory parameters to be established. One group of rats received vehicle (saline, 1 ml/kg, IV; n = 9 rats; 326 ± 2 g) and another received GSHee (100 μmol/kg, IV; n = 9 rats; 329 ± 2 g). After 15 min, all rats received an injection of fentanyl (75 μg/kg, IV). The effects of bolus injections of GSH (100 μmol/kg, IV; 324 ± 3 g; n = 9 rats) or vehicle (saline; 326 ± 2 g; n = 9 rats) on fr, Vt and Ve were determined in freely-moving male Sprague–Dawley rats and the subsequent effects of fentanyl (75 μg/kg, IV) given 15 min were also determined in both groups of rats. Due to the closeness of the body weights of the two groups of rats, ventilatory data are shown without body weight corrections. Parameters were breathing frequency (fr), tidal volume (Vt), minute ventilation (Ve), inspiratory time (Ti), expiratory time (Te), peak inspiratory (PIF) and peak expiratory (PIF) flows. The provided software (Fine Pointe, BUXCO) constantly corrected digitized values for changes in chamber temperature and humidity. Pressure changes associated with the respiratory waveforms were then converted to volumes (i.e., Vt, PIF and PEF) using the algorithm of Epstein and colleagues^98,99^. Specifically, factoring in chamber temperature and humidity, the cycle analyzers filtered the acquired signals, and BUXCO algorithms (Fine Pointe) generated an array of box flow data that identified a waveform segment as an acceptable breath. From that data vector, the minimum and maximum values were determined. Flows at this point were considered to be “box flow” signals. From this array, the minimum and maximum box flow values were determined and multiplied by a compensation factor provided by the selected algorithm^98,99^, thus producing Vt, PIF and PEF values that were used to determine accepted and rejected waveforms reported as Rejection Index (non-eupneic breathing)^97^. All directly recorded parameters including Rejection Index (see below) were extracted from the raw waveforms using Data Sciences International (St. Paul, MN, USA) proprietary Biosystem XA software (version 2.9.0.2) and proprietary FinePointe software (version v2.8.0), as described previously^87–97^ and as detailed in the Data Sciences International/Buxco website reference to the list of parameters provided by FinePointe Software using whole body plethysmography (https://www.datasci.com/products/buxco-respiratory-products/finepointe-whole-body-plethysmography). The BioSystem XA software extracts the waveforms that are analyzed by the FinePointe software that uses National Instruments Measurement Studio to perform these analyses (http://zone.ni.com/reference/en-XX/help/372636F-01/mstudiowebhelp/html/5d5b3031/).

### Righting reflex

Separate groups of adult male Sprague–Dawley rats were used to evaluate the effects of GSHee (100 μmol/kg, IV) on the duration of fentanyl (75 μg/kg, IV)-induced impairment of the righting reflex (inability to stand on all four legs). Each rat was placed in an open plastic chamber to allow the duration of the loss of righting reflex to be accurately recorded. The time at which the rat spontaneously stood on all four paws was taken as the point of recovery^100–102^. In this study, one group of rats (320 ± 2 g, n = 12) received an injection of vehicle (saline) and after 15 min, an injection of fentanyl. A second group of rats (323 ± 2 g, n = 12) received an injection of GSHee and after 15 min an injection of fentanyl. The duration of effect of fentanyl was defined as the time interval from the time of injection of fentanyl administration to the recovery of righting reflex.

### Statistics

The recorded data (1 min bins) and derived parameters, Vt/Ti and Response Area (cumulative percent changes from pre-values) were taken for statistical analyses. The pre-drug 1 min bins excluded occasional marked deviations from resting due to movements or scratching by the rats. These exclusions ensured accurate determinations of baseline parameters. The data are presented as mean ± SEM. All data were analyzed by one-way or two-way analysis of variance followed by Student's modified *t* test with Bonferroni corrections for multiple comparisons between means using the modified error mean square term (EMS) from the ANOVA^103^. The modified *t-*statistic is t = (mean group 1—mean group 2)/[s x (1/n_1_ + 1/n_2_)^1/2^] where s^2^ = the mean square within groups term from the ANOVA (the square root of this value is taken for the modified t-statistic formula) and n_1_ and n_2_ are the number of rats in each group. Based on an elementary inequality called Bonferroni’s inequality, a conservative critical value for the modified *t*-statistics is obtained from tables of *t*-distribution using a significance level of P/m, where m is the number of comparisons between groups to be performed. The degrees of freedom are those for the mean square for within group variation from the ANOVA table. In most cases, the critical Bonferroni value cannot be obtained from conventional tables of the t- distribution but may be approximated from widely available tables of the normal curve by t* = z + (z + z^3^)/4n, where n is the degrees of freedom and z is the critical normal curve value for P/m^104^. As demonstrated by Wallenstein et al.^103^ the Bonferroni procedure is recommended for general use since it is easiest to apply, has the widest range of applications, and gives critical values that will be lower than those of other procedures if the investigator is able to limit the number of comparisons, and that will be only slightly larger than those of other procedures if many comparisons are made. A value of *P* < 0.05 was taken as the initial level of statistical significance^103,104^.

## Results

### Tail-flick latencies

**Figure 1:**
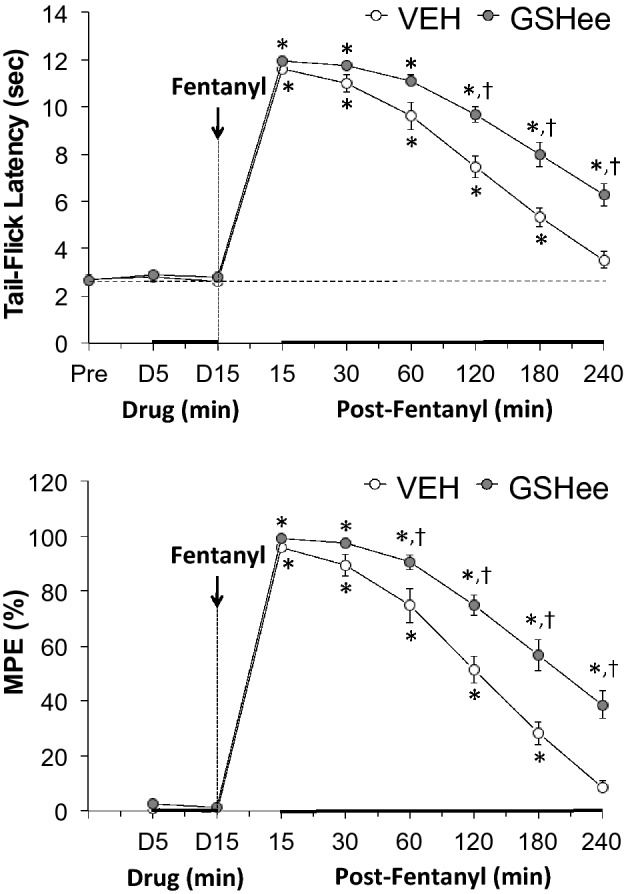
Upper panel: Effects of fentanyl (75 μg/kg, IV) on tail-flick latencies in rats pretreated with vehicle (VEH; 1 ml/kg, IV) or GSHee (100 μmol/kg, IV). Lower panel: Data in the upper panel expressed as maximal possible effect (MPE, %). All data are presented as mean ± SEM. There were 9 rats in each group. The data were analyzed by repeated measures ANOVA followed by multiple comparison testing as detailed in the Methods section. *P < 0.05/6 comparisons per group, significant change from post-drug (i.e., 15, 30, 60, 120, 180 or 240 min post-fentanyl versus D15 value). ^†^*P* < 0.05/6 between group comparisons, GSHee *versus* vehicle.

As summarized in Fig. 1, a bolus injection of GSHee (100 μmol/kg, IV) or vehicle (VEH, IV) did not alter TFL as measured 5 and 15 min after administration. The subsequent injection of fentanyl (75 μg/kg, IV) elicited robust antinociception for at least 180 min in vehicle-treated rats. As also seen in Fig. 1, the duration of the fentanyl-induced antinociception was greater in GSHee-pretreated rats than in the vehicle-pretreated rats.

### Arterial blood-gas chemistry and A-a gradient

**Figure 2:**
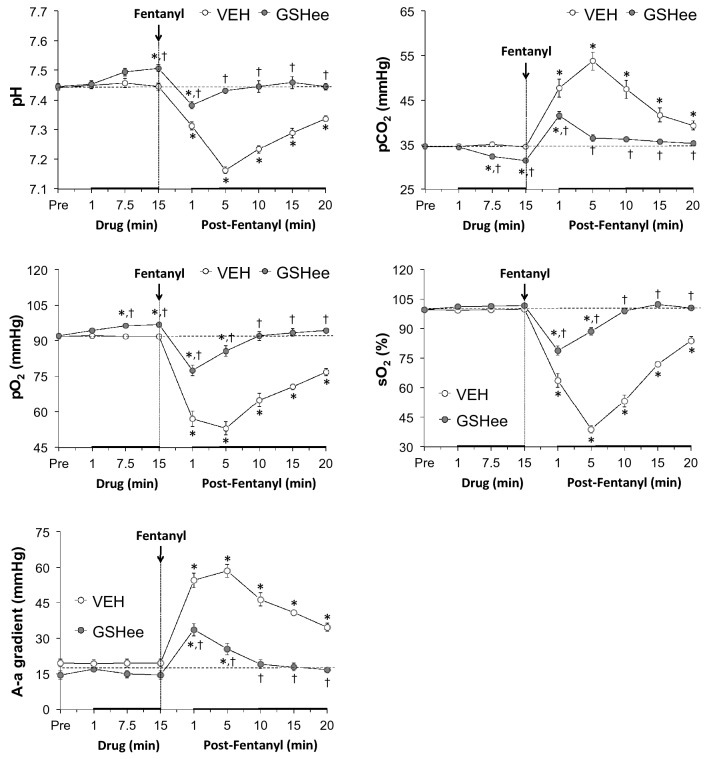
Effects of fentanyl (75 μg/kg, IV) on arterial blood pH, pO_2_, pCO_2_ and sO_2_ values and Alveolar-arterial (A-a) gradients in rats pretreated with vehicle (VEH; 1 ml/kg, IV) or GSHee (100 μmol/kg, IV). The data are presented as mean ± SEM. The data were analyzed by repeated measures ANOVA followed by multiple comparison testing as detailed in the Methods section. There were 9 rats in each group. *P < 0.05/5 comparisons per group, significant change from post-drug. ^†^*P* < 0.05/5 between group comparisons, GSHee *versus* vehicle.

As summarized in Fig. 2, the injection of GSHee (100 μmol/kg, IV) elicited minor but significant increases in pH and pO_2_ that were accompanied by a minor but significant decrease in pCO_2_. These responses were still evident 20 min after injection of fentanyl. GSHee did not affect sO_2_ or A-a gradient. The injection of fentanyl (75 μg/kg, IV) in vehicle-treated rats elicited dramatic decreases in arterial blood pH, pO_2_ and sO_2_ that were accompanied by substantial increases in pCO_2_ and A-a gradient (Fig. 2). The changes in ABG chemistry and A-a gradient were markedly smaller in magnitude and of substantially shorter duration in the GSHee-pretreated rats.

### Body temperature

The changes in BT elicited by injection of fentanyl (75 μg/kg, IV) in vehicle-treated or GSHee (100 μmol/kg, IV)-treated rats are summarized in Supplemental Table 1. Prior to the injections, both groups had similar resting BT values. Neither vehicle nor GSHee affected BT as recorded at 5 and 15 min post-injection. The injection of fentanyl in vehicle-treated rats elicited a small but significant hyperthermia. Fentanyl elicited a similar minor hyperthermia in the GSHee-treated rats.

### Ventilatory parameters

**Figure 3:**
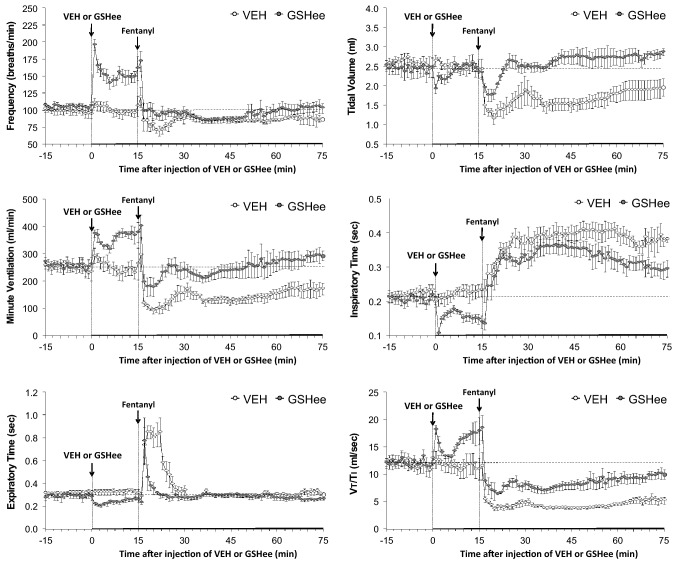
Effects of fentanyl (75 μg/kg, IV) on frequency of breathing (top left panel), tidal volume (top right panel), inspiratory time (middle left panel), minute ventilation (middle right panel), expiratory time (bottom left panel) and tidal volume/inspiratory time (Vt/Ti) in rats pretreated with vehicle (VEH; 1 ml/kg, IV) or GSHee (100 μmol/kg, IV). The data are presented as mean ± SEM. There were 9 rats in each group. The stippled horizontal line denotes average resting values before injection of GSHee or vehicle.

As summarized in Fig. 3, the injection of GSHee elicited a prompt and sustained increase in breathing frequency (fr) that was accompanied by expected decreases in inspiratory time (Ti) and expiratory time (Te). GSHee did not affect tidal volume (Vt) and so the sustained increase in minute ventilation (Ve) was due entirely to the increase in fr. The changes in Vt and Ti resulted in a biphasic increase in inspiratory drive (Vt/Ti). The injection of vehicle did not alter any of the above ventilatory parameters. Subsequent injection of fentanyl in vehicle-treated rats elicited (a) a decrease in fr associated with a pronounced and sustained increase in Ti and a pronounced but shorter in duration increase in Te, and (b) sustained decreases in Vt, Ve and inspiratory drive (Vt/Ti). Subsequent injection of fentanyl in GSHee-treated rats elicited (i) a substantially smaller decrease in fr that was accompanied by a substantially smaller increase in Te but a smaller reduction of the increase in Ti, and (ii) smaller decreases in Vt, Ve and inspiratory drive.

**Figure 4:**
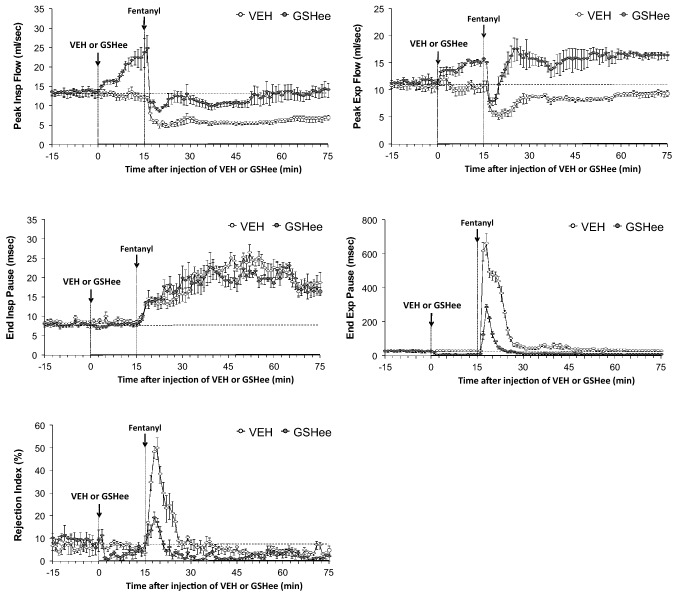
Effects of fentanyl (75 μg/kg, IV) on peak inspiratory flow (top left panel), peak expiratory flow (top right panel), end inspiratory pause (middle left panel), end expiratory pause (middle right panel), and Rejection Index (bottom left panel) in rats pretreated with vehicle (VEH; 1 ml/kg, IV) or GSHee (100 μmol/kg, IV). The data are presented as mean ± SEM. There were 9 rats in each group. The stippled horizontal line denotes average resting values before injection of GSHee or vehicle.

As shown in the top panels of Fig. 4, GSHee elicited robust increases in peak inspiratory flow (PIF) and peak expiratory flow (PEF) that were still present at the time fentanyl was injected 15 min later. Fentanyl elicited pronounced and sustained decreases in PIF and PEF in the vehicle-treated rats. The injection of fentanyl elicited a robust and sustained decrease in PIF in the GSHee-treated rats but the levels of PIF did not fall markedly below baseline (pre-GSHee levels). In contrast, fentanyl elicited a transient reduction in PEF with the levels quickly returning and remaining at the elevated levels elicited by GSHee. As shown in the middle panels of Fig. 4, GSHee elicited robust decreases in end expiratory pause (EEP), but only minor changes in end inspiratory pause (EIP) (more readily seen in Supplemental Fig. 1). The subsequent injection of fentanyl elicited pronounced and sustained increases in EIP that were similar in the vehicle- and GSHee-treated rats. In contrast, fentanyl elicited a marked increase in EEP for approximately 25 min in the vehicle-treated rats, but a markedly smaller response of lesser duration in the GSHee-treated rats. Finally, the bottom panel of Fig. 4 shows that GSHee elicited a fall in Rejection Index (Rinx, decrease in non-eupneic breathing) that had transpired by the time fentanyl was given. The subsequent injection of fentanyl elicited a marked increase in Rejection Index for approximately 15 min in vehicle-treated rats, but a markedly smaller increase of lesser duration in GSHee-treated rats. Rejection Index tended to drop in both groups of fentanyl-injected rats, most noticeably about 30 min after injection of fentanyl (45 min after injection of vehicle or GSHee).

Supplemental Fig. 1 summarizes the *cumulative* responses elicited by injections of vehicle (VEH) or GSHee (top panel) recorded over the 15 min-period prior to injection of fentanyl and those elicited by subsequent injection of fentanyl recorded for 60 min (bottom panel). The injection of vehicle did not elicit cumulative changes in any ventilatory parameter. GSHee elicited cumulative increases in Ve, which was due solely to increases in fr not Vt. GSHee elicited cumulative decreases in Ti and Te; cumulative increases in inspiratory drive (Vt/Ti), PIF, and PEF; no cumulative change in EIP, but cumulative decreases in EEP and Rejection Index. The subsequent injection of fentanyl in the vehicle-treated rats (bottom panel) elicited cumulative decreases in fr, Vt and Ve; cumulative increases in Ti and to a lesser extent Te; cumulative decreases in PIF and PEF; and cumulative increases in EIP, EEP and Rejection Index. Pretreatment with GSHee prevented/attenuated/reversed the fentanyl-induced cumulative decreases in Vt, Ve, Vt/Ti, PIF, PEF, and fentanyl-induced cumulative increases in EIP, EEP and Rejection Index. We also investigated whether pre-treatment with GSH (100 μmol/kg, IV) itself would modulate the ventilatory depressant effects of fentanyl (75 μg/kg, IV). The baseline (Pre) fr, Vt and Ve values in the two groups of rats used in this study are presented in Supplemental Table 3. There were no-between group differences in any parameter. The changes in fr, Vt and Ve elicited by the injection of vehicle (VEH, saline) or GSH and the subsequent responses elicited by fentanyl are summarized in Supplemental Fig. 2. As can be seen, the injection of GSH elicited a prompt but short-lived increase in Freq with minor effects on TV that resulted in short-lived increases in MV. The responses elicited by the subsequent injection of fentanyl were similar in the vehicle- or GSH-treated rats.

### Behaviors

Despite the relatively pronounced increase in Freq in the rats that received GSHee, the rats that did not display any obvious behavioral signs (e.g., movement, squealing, scratching or sniffing) and the rats that were lying quietly at the time of injection, remained so immediately during and following the injection. In addition, the duration of the pronounced sedation elicited by fentanyl was very similar in vehicle- or GSHee-treated rats. More specifically, we determined that in other groups of adult male Sprague–Dawley rats that injection of fentanyl (75 μg/kg, IV) caused immediate sedation (the rats quickly become immobile and lay on their side with eyes most often closed).The full return of the righting-reflex in vehicle-treated rats (43 ± 6 min, n = 12) and GSHee (100 μmol/kg, IV)-treated rats (54 ± 9 min, n = 12) were similar to one another (*P* > 0.05, GSHee *versus* vehicle).

### Discussion

The intravenous injection of 100 μmol/kg (33.54 mg/kg) of GSHee elicited an array of responses in freely-moving adult male Sprague–Dawley rats and had dramatic effects on the responses elicited by subsequent injection of fentanyl (100 μmol/kg). It is likely that GSHee exerts its effects in naïve rats by increasing levels of GSH and metabolites (γ-glutamylcysteinyl and cysteine) in cells (e.g., neurons) in which GSH^53–74^ and the metabolites^82,105–108^ affect a number of biological processes. It is unlikely that GSHee directly inhibits OR function (loss of affinity or down-regulation of membrane accessible receptors) since GSHee augmented the analgesic effects of fentanyl, which are known to be OR-mediated^15^. It is well known that morphine can markedly lower GSH and cysteine levels in brain and peripheral tissues^109–117^. To our knowledge, there is no literature describing the effects of fentanyl on tissue GSH levels. However, one study reported that fentanyl causes an increase in production of reactive oxygen species in freshly isolated peripheral blood lymphocytes^118^, which arguably may involve a reduction in cellular reduced thiol levels. It must be noted that it is far from clear how a proposed fentanyl-induced decrease in intracellular GSH and cysteine levels would contribute to fentanyl-induced depression of ventilation. Moreover, it is not clear whether the ability of GSHee to blunt the ventilatory depressant effects of fentanyl is due to enhancement of GSH and cysteine levels in relevant tissues such as the brainstem and carotid bodies. It should also be noted that the ability of fentanyl to enhance the production of reactive oxygen species in lymphocytes is far from definitive with respect to how fentanyl depresses ventilation or how GSHee modulates the effects of fentanyl. The finding that the injection of GSH itself did not blunt the ventilatory depressant effects of fentanyl raises the tentative suggestion that the bioactivity of GSHee may be due to the enhanced ability of the thiolester to enter cells that control breathing and that this intracellular entry is related to the sustained efficacy of GSHee against some of the actions of fentanyl.

With respect to analgesia, GSHee did not elicit observable changes in analgesia status of freely moving rats per se whereas the duration of fentanyl analgesia was increased in rats pretreated with the thiolester. The mechanisms by which GSHee augments fentanyl-induced analgesia are probably multi-factorial and likely to involve modulation of analgesia-signaling pathways within the periphery, spinal cord and brain^15^. Indeed, it may be possible that GSHee modulates opioid receptor signaling processes that result in fentanyl becoming a “biased” ligand so that the opioid receptor signaling pathways now favor analgesia rather than respiratory depression^119,120^. There is no literature on whether GSH or GSHee can alter the bias of fentanyl-induced signaling but molecular studies that look at the effects of GSH and GSHee on the interaction between ligand-occupied opioid receptors and coupling of Gproteins and b-arrestins for example, would seem to be feasible^119,120^. Whatever the mechanism, opioid-induced analgesia augmentation is a very important attribute for an OIRD drug candidate. The dose of fentanyl we chose for this study (75 μg/kg, IV) elicited a robust decrease in minute ventilation (approximately 50% over a period of at least 60 min) and so represents a substantial level of ventilatory impairment by which to examine the effects of the 100 μmol/kg dose of GSHee. However, it is clear that the effects of lower and higher doses of GSHee need to be evaluated against higher doses of fentanyl that are known to cause a more dangerous depression of breathing^121^. This is to fully characterize the full efficacy of GSHee but also to take into account recent evidence from elegant in situ brainstem studies showing that morphine affects key areas of the respiratory network in a dose-dependent manner^122^.

The ability of GSHee to affect ventilatory parameters in naïve rats was as complex as it was impressive. First, GSHee elicited a sustained increase in Ve via the sustained increase in fr that was accompanied by the expected decreases in both Ti and Te, whereas it had minimal effects on Vt. It would seem likely that GSHee drives fr by actions within the carotid bodies by mechanisms including direct effects on neurotransmitter release from primary glomus cells. However, Gonzalez et al.^123^ provided evidence that GSH did not activate chemoreceptor cells in normoxia or modify hypoxic activation of these cells even though incubation of carotid body glomus cells with reduced GSH increased GSH-reducing potential. It remains possible that nitrosyl derivatives of GSHee such as S-nitrosoglutathione and S-nitrosocysteinylglycine or S-nitrosocysteine drive an increase in fr by activating carotid body glomus cells, chemoafferent terminals in the carotid bodies, or by actions in brain sites such as the nucleus tractus solitarius^40,124^, a key nucleus of ventilatory processing. Despite minimal effects on Vt (actual volume of air taken in each breath), GSHee did elicit a sustained increase in both PIF and PEF, which suggests the thiolester either directly affected the force of contraction of skeletal muscle in the extrinsic (inspiratory) and intrinsic (expiratory) intercostals and/or diaphragm. This would be consistent with considerable in vitro evidence that GSH enhances both Ca^2+^-dependent and Ca^2+^-independent skeletal muscle contractility^125–127^.

As we reported previously^15^, the injection of fentanyl elicits much longer increases in Te than Ti in vehicle-treated rats. As such, it would appear that the effects of fentanyl on the processes driving active inspiration in intact rats are of much longer duration than those that occur during passive expiration. Saunders and Levitt^128^ provided elegant demonstration that the fentanyl-induced changes in Te and Ti occurred in parallel in the in situ arterially-perfused rat brainstem preparation. We therefore presume that sensory inputs or other mechanisms not available in the in situ presentation are responsible for the exaggerated effects on Ti as opposed to Te in vivo. The mechanisms by which fentanyl exerts its longer-lasting effects on Ti remain to be determined. The effects on GSHee on the fentanyl-induced changes in Te (GSHee dramatically curtailed the effects of fentanyl on Te) were perhaps more remarkable than they were on Ti (GSHee somewhat diminished the magnitude rather than the duration of the longer lasting elevation in Ti). We cannot speculate on how GSHee elicits these effects but it is clear that it is available to interfere with the processes by which fentanyl suppresses active and passive phases of breathing. Fentanyl would be expected to cause non-eupneic breathing with more variation in phase length as the respiratory rate drops^122,129^. Indeed, in the present study we found that the relative increases in Ti and Te were substantially different during the peak decreases in fr elicited by fentanyl (approximately + 5 min post-injection, see Fig. 3). As can be seen in Supplementary Table 4, Te lengthened considerably more than Ti at the + 5 min time-point such that the Ti/Te ratio fell dramatically (-37 ± 5%). Moreover, at the 30 min post-fentanyl time-point when fr was close to return to pre-values (see Fig. 3), Ti was still substantially longer than pre-fentanyl whereas Te had returned to pre-values so that the Ti/Te ratio rose dramatically (+ 81 ± 9%). As also seen in Fig. 3, Ti values were substantially lower at the + 5 and + 30 min time-points post-fentanyl in the GSHee-treated rats whereas Te values were equivalent to pre-values at both times, reinforcing the general observation that GSHee differentially effects the ventilatory effects of fentanyl.

Under physiological circumstances, the ratio of Vt/Ti is an accepted index of inspiratory drive although it could be argued that changes in PIF could be taken as a similar index of changes in inspiratory drive^14^. However, the changes in Vt/Ti and PIF elicited by GSHee were not exactly the same (GSHee caused an initial transient spike in Vt/Ti not seen in PIF) although Vt/Ti and PIF both rose steadily between 5 to 15 min following injection. The injection of fentanyl elicited robust and long-lasting decreases in both Vt/Ti and PIF in vehicle-treated rats. However, it is clear that the depressant effects of fentanyl on PIF in the GSHee-treated rats were substantially less than the effects on Vt/Ti. As such, one possible interpretation of these findings is that GSHee does not entirely blunt the processes by which fentanyl suppresses inspiratory drive (Vt/Ti) it does have more pronounced effects on the processes by which fentanyl suppresses PIF.

Another compelling finding was that GSHee markedly reduced the occurrence of non-eupneic breaths (usually expressed as apneas, type 1 and 2 sighs) despite actually increasing fr, which usually is a stimulus to enhance non-eupneic breathing^87^. This would tentatively suggest that GSHee exerts positive effects in brain regions such as the Kölliker-Fuse and pre-Bötzinger complex, which generate/control rhythmic breathing^121–132^, although to our knowledge there is no reports as to the direct effects of GSH in these neuronal complexes. With respect to the rejection index, it must be noted that the summation of sighs and apneas under non-eupneic breathing under the rejection index is obviously simplistic and does not do justice to the different mechanisms that elicit these individual respiratory patterns. Sighs are reported to result from activation of a neuronal circuit in the preBötzinger complex/parafacial group^133^ while apnea results from direct inhibition of the preBötzinger complex or inhibition of drive to phase-switching neurons in the preBötzinger complex^134^. An increase in respiratory drive elicited by GSGee may decrease the number of sighs and decrease the number of apneas, but that would not be defined definitively by a change in the rejection index since this index also includes sniffs and changes in respiratory waveforms due to behavioral movements^87^, although it must be noted that the fentanyl-treated rats (those that received vehicle or GSHee) were basically immobile throughout the ventilatory study. The ventilatory effects of GSHee resulted in predictable (minor) changes in ABG chemistry including an increase in pH, a decrease in pCO_2_ and an increase in pO_2_. There was minimal change in A-a gradient suggesting that GSHee did not directly affect gas-exchange processes in the lungs.

The injection of fentanyl to vehicle (saline)-pretreated rats elicited a sustained reduction in fr that was associated with pronounced long-lasting increases in Ti and EIP (at least 60 min) and pronounced but shorter-lived increases in Te and EEP (about 15 min in duration). Fentanyl also elicited a marked and sustained (at least 60 min) decrease in Vt (coupled to the decrease in fr resulting in a marked decrease in Ve), inspiratory drive (Vt/Ti), PIF and PEF. Finally, fentanyl caused a marked increase in Rejection Index (elevated disordered breathing) for 10–15 min. The potential sites and mechanisms of action underlying these effects of fentanyl have been addressed in detail elsewhere^11–15^. Consistent with our previous study^15^, the injection of fentanyl was associated with minor increases in BT that would have minimal direct effects on breathing.

Our major findings were that the ventilatory depressant effects and negative changes in ABG chemistry and A-a gradient elicited by fentanyl were markedly attenuated in rats pretreated with GSHee. With respect to fr, it would be easy to conclude that fentanyl still exerts dramatic effects since fentanyl elicited a rather precipitous fall in fr even though the values did not fall below baseline values. GSHee had little effect on the increase in Ti elicited by fentanyl. However, GSHee had a dramatic impact on the duration of fentanyl-induced increase in Te. Indeed, the substantially smaller fentanyl-induced increase in Te in the presence of GSHee suggests that GSHee enhances the central processes driving the end of expiration (inspiratory on-switch) and thus decrease expiratory duration. In addition, the thiolester may directly combat the ability of fentanyl to suppress the central processes driving expiration under fentanyl-induced hypoxic/hypercapnic conditions and/or the direct inhibitory effects of fentanyl on expiratory muscle activity^11–15^, although it should be noted that there is evidence that opioids can actually increase expiratory muscle activity under certain circumstances^135^. The effects of GSHee on the ability of fentanyl to lower PIF and PEF may also provide some discrimination as to potential sites and mechanisms of action of the thiolester. Remembering that GSHee elicited sustained increases in PIF and PEF, it was evident that fentanyl was able to elicit strong reductions in these parameters. The difference was that PIF fell to levels approximately equivalent to baseline values (before any drug was given) whereas the fentanyl-induced reduction in PEF was short-lived and PEF rapidly returned to the elevated levels seen after injection of GSHee. As such, it is possible that GSH-dependent processes may be more influential on central and peripheral mechanisms driving expiratory chest-muscles than inspiratory chest-muscles.

Opioid analgesics (e.g., fentanyl, morphine, etorphine, buprenorphine, methadone, butorphanol, oxymorphone) have been demonstrated to increase A-a gradient in a variety of species including humans^136–138^, goats^139^, rabbits^140^, dogs^141^, impala^142^, and rats^15,94,95^. As discussed by Meyer et al.^139^, and Henderson et al.^15^, opioids increase A-a gradients by impairing ventilation-perfusion ratios in the lungs (ventilation-perfusion mismatch). For example, opioids decrease pulmonary perfusion via hypoxia-induced pulmonary vasoconstriction^143^, and by direct pulmonary vasoconstriction^144,145^ by mechanisms including centrally-mediated activation of sympathetic nerve activity to the lungs^146^, and induction of histamine release in the lungs^144,147^. As such, the ability of GSHee to overcome the fentanyl-induced increase in A-a gradient is most likely due to the direct GSHee-induced increase in Ve and potentially by interfering with any of the other mechanisms described immediately above.

The possibility that GSHee directly blunted the mechanisms by which fentanyl depressed breathing is supported by the evidence that GSHee did not affect baseline Vt values but markedly blunted the magnitude and duration of fentanyl-induced reduction fentanyl-induced reduction in Vt. This effect on Vt suggests that GSHee or its functional metabolites/nitrosylated species block central and peripheral signaling pathways by which fentanyl suppresses Vt^15^. This key effect of GSHee is connected to the findings that GSHee reduced the ability of fentanyl to lower pH, pO_2_ and sO_2_ while elevating pCO_2_ (all consistent with a fall in Ve) and blunted the fentanyl-induced increase A-a-gradient (i.e., impaired gas-exchange in the lungs). The ability GSHee to suppress fentanyl-induced disordered (non-eupneic) breathing raises the possibility that GSH-dependent mechanisms in key brainstem sites responsible for the control of breathing may be recruited to prevent the negative effects of fentanyl on eupneic breathing. An important consideration with respect to the ability of GSHee to prevent the deleterious effects of fentanyl on breathing was that the opioid appeared to elicit its full sedative effects in the presence of the GSHee (e.g., righting-reflex times and general observations of behavior could not discriminate between the effects of fentanyl in vehicle-treated or GSHee-treated rats). It could be expected that abrupt arousal may have led to enhanced breathing but as this did not appear to happen it is likely that GSHee interacted directly with the neural pathways/cellular mechanisms responsible for fentanyl-induced OIRD.

In summary, GSHee and L-CYSee^21^ may represent the first examples of a novel class of thiolester compounds that are able to effectively prevent the ventilatory depressant effects of powerful opioids such as fentanyl (and perhaps other high potency opioids such as sufentanil and alfentanil) and their deleterious effects on breathing stability and gas-exchange within the lungs without compromising the analgesic actions of the opioids. The obvious caveat is that we have only tested GSHee against a 75 mg/kg dose of fentanyl, that while eliciting a substantial degree of ventilatory depression, it does not approach the lethality of higher doses of this opioid. The ability of GSHee to prevent the negative effects of fentanyl on Vt is a key factor in the therapeutic potential of this thiolester in the treatment of OIRD. However, the vital importance of the ability of GSHee to overcome the negative effects of fentanyl on gas-exchange in the lungs cannot be over-emphasized. The administration protocol used in this study highlights the ability of GSHee to affect ventilatory parameters and to blunt the negative effects of fentanyl on breathing. It would certainly be preferable that GSHee be delivered in a dose form that would not elicit profound responses prior to administration of an opioid and we are establishing whether lower doses or a constant infusion can achieve blockade of fentanyl-induced OIRD without eliciting dramatic effects on ventilation prior to administration of the opioid. For reasons that are obvious in terms of clinical use and the current opioid crisis, we are also establishing whether GSHee can elicit a prompt and sustained reversal of the negative effects of opioids without eliciting unwanted side-effects such as hyperventilation.

Pretreatment with GSHee blunted the effects of the subsequent injection of fentanyl in a manner that suggest that the actions are heavily dependent on the actions of fentanyl itself on some parameters but not others. More specifically, pretreatment with GSHee elicited a sustained attenuation of the long-lasting negative effects of fentanyl on Vt, Vt/Ti and PIF whereas it had relatively minimal effects on the long-lasting effects of fentanyl on Ti and EIP. In addition, pretreatment with GSHee elicited a pronounced attenuation of the relatively short-lived effects of fentanyl on fr and Te, EEP and Rejection Index. As such, it is evident the GSHee reaches the systems/circuitry that are involved in some of the actions of fentanyl but not others. A search of the literature has not yielded any information as to whether GSH/GSHee may be a ventilatory stimulant in humans and/or whether they blunt the ventilatory depressant effects of opioids. As such, the key question remains as to whether the efficacy of GSHee in rats will be translated into humans.

GSHee could be an addition to the important strategy to mitigate OIRD via co-treatment with non-OR ventilatory stimulants that do not affect opioid-induced analgesia. Several classes of non-OR ventilatory stimulants are currently being investigated with many acting in the brainstem respiratory network including D1-dopamine receptor agonists, 5-hydroxytryptamine receptor modulators, α-amino-3-hydroxy-5-methyl-4-isoxazolepropionic acid receptor agonists (ampakines), thyrotropin-releasing hormone, the endogenous peptide glycyl-glutamine, and phospodiesterase-4 inhibitors. Others include doxapram and GAL021 that act on K^+^-channels on O_2_-sensing cells of the carotid bodies. In their definitive review, Dahan et al.^148^ critically appraised the efficacy of these ventilatory stimulants and concluded that “none of the experimental drugs are adequate for therapeutic use in OIRD and all need further study of efficacy and toxicity. Nonetheless, Dahan et al.^148^ did point out that all of these drugs “highlight potential mechanisms of action and possible templates for further study and development”. We believe that GSHee could be added to this exciting list.

## Supplementary Information

Supplementary Information.
