# Supplementary material for: Glutathione ethyl ester reverses the deleterious effects of fentanyl on ventilation and arterial blood-gas chemistry while prolonging fentanyl-induced analgesia
Source: Sci Rep. 2021 Mar 26;11:6985. doi: 10.1038/s41598-021-86458-x (PMC7997982; doi:10.1038/s41598-021-86458-x)
Supplement: Supplementary file 1 — Supplementary Information. [file 41598_2021_86458_MOESM1_ESM.docx]

**Supplement**

**Glutathione ethyl ester reverses the deleterious effects of fentanyl on ventilation and arterial blood-gas chemistry while prolonging fentanyl-induced analgesia**

Michael W. Jenkins,^1,2^ Faiza Khalid,^3^ Santhosh M. Baby,^4,^**^†^** Walter J. May,^5^ Alex P. Young,^5^

James N. Bates,^6^ Feixiong Cheng,^7^ James M. Seckler,^1^ Stephen J. Lewis^2,8,^*

*^1^Department of Biomedical Engineering, Case Western Reserve University, Cleveland, Ohio, USA*

*^2^Department of Pediatrics, Case Western Reserve University, Cleveland, Ohio, USA*

*^3^Department of Internal Medicine, University Hospitals, Case Western Reserve University, Cleveland, Ohio, USA*

*^4^Section of Biology, Galleon Pharmaceuticals, Inc., Horsham, Pennsylvania, USA*

*^5^Department of Pediatrics, University of Virginia, Charlottesville, Virginia, USA*

*^6^Department of Anesthesia, University of Iowa, Iowa, USA*

*^7^Cleveland Clinic Lerner College of Medicine, Case Western Reserve University, Cleveland, OH, USA*

*^8^Department of Pharmacology, Case Western Reserve University, Cleveland, Ohio, USA*

**^†^Current address:** Santhosh M. Baby, Translational Sciences Treatment Discovery, Galvani Bioelectronics, Inc., 1250 S Collegeville Rd., Collegeville, Pennsylvania 1r9426. Email: santhosh.m.baby@galvani.bio

***Address correspondence to:** Stephen J. Lewis, PhD. Department of Pediatrics, Case Western Reserve University, 10900 Euclid Avenue, Cleveland, OH 44106-4984, USA. Telephone: 216-368-3482. Fax: 216-368-4223. Email: [sjl78@case.edu](mailto:sjl78@case.edu)

**Supplemental Table 1**

Effects of fentanyl (75 μg/kg, IV) on body temperature in vehicle- or GSHee (100 μmol/kg, IV)-treated conscious rats

|  |  | **Treatment Groups** | | |
| --- | --- | --- | --- | --- |
| **Phase** | **Time (min)** | **Vehicle** |  | **GSHee** |
| **Pre** | 30 | 37.8 ± 0.1 |  | 37.7 ±0.1 |
|  | 15 | 37.9 ± 0.1 |  | 37.8 ± 0.1 |
|  | 5 | 37.8 ± 0.1 |  | 37.8 ± 0.1 |
|  | **Average** | 37.8 ± 0.1 |  | 37.8 ± 0.1 |
| **Post-drug** | 5 | 37.8 ± 0.1 |  | 37.9 ± 0.1 |
|  | 15 | 37.9 ± 0.1 |  | 37.9 ± 0.1 |
| **Post-fentanyl** | 5 | 37.8 ± 0.1 |  | 37.9 ± 0.1 |
|  | 10 | 38.0 ± 0.1 |  | 38.0 ± 0.1 |
|  | 15 | 38.0 ± 0.1 |  | 38.0 ± 0.1 |
|  | 20 | 38.1 ± 0.1 |  | 38.2 ± 0.1 |
|  | 25 | 38.2 ± 0.1 |  | 38.2 ± 0.1 |
|  | 30 | 38.1 ± 0.1 |  | 38.2 ± 0.1 |
| **Change from Pre (^o^C)** | 5 | -0.02 ± 0.10 |  | 0.06 ± 0.09 |
|  | 10 | 0.13 ± 0.09 |  | 0.17 ± 0.11 |
|  | 15 | 0.09 ± 0.08 |  | 0.13 ± 0.10 |
|  | 20 | 0.21 ± 0.08* |  | 0.31 ± 0.09* |
|  | 25 | 0.29 ± 0.05* |  | 0.33 ± 0.10* |
|  | 30 | 0.23 ± 0.08* |  | 0.31 ± 0.08* |

The data are presented as mean ± SEM. There were 9 rats in each group. **P* < 0.05, significant change from Pre. There were no between-group differences in the effects of fentanyl (P > 0.05/6 comparisons at 5, 10, 15, 20, 25 and 30 min, for all between-group comparisons).

**Supplemental Table 2**

Baseline respiratory values prior to administration of any drugs

| **Parameter** | **Vehicle** | **GSHee** |
| --- | --- | --- |
| Number of rats | 9 | 9 |
| Frequency, breaths/min | 98 ± 5 | 104 ± 5 |
| Tidal volume, ml | 2.56 ± 0.12 | 2.44 ± 0.20 |
| Minute Ventilation, ml/min | 249 ± 15 | 254 ± 18 |
| Inspiratory Time, sec | 0.218 ± 0.013 | 0.206 ± 0.015 |
| Expiratory Time, sec | 0.311 ± 0.021 | 0.285 ± 0.023 |
| Tidal Volume/Respiratory Time, ml/sec | 12.0 ± 1.0 | 11.9 ± 0.6 |
| End Inspiratory Pause, msec | 8.3 ± 0.3 | 7.7 ± 0.4 |
| End Expiratory Pause, msec | 22.1 ± 1.7 | 24.1 ± 0.8 |
| Peak Inspiratory Flow, ml/sec | 13.2 ± 0.7 | 13.4 ± 0.8 |
| Peak Expiratory Flow, ml/sec | 10.6 ± 0.4 | 11.3 ± 0.5 |
| Rejection Index (%) | 6.5 ± 0.9 | 8.8 ± 1.8 |

The data are presented as mean ± SEM. There were 9 rats in each group. There were no between-group differences for any resting parameter (P > 0.05, for all comparisons).

**Supplemental Figure 1**

**Supplemental Figure 1.** **Top panel.** Cumulative ventilatory responses (expressed as %change from pre-values) elicited by the injections of vehicle (VEH; 1 ml/kg, IV) or GSHee (100 μmol/kg, IV) recorded over the 15 min period immediately prior to injection of fentanyl. The 15 values were added together to obtain the cumulative response for each rat and the mean and SEM of these values from the 9 rats was calculated. **Bottom panel:** Cumulative ventilatory responses (expressed as %change from pre-values) elicited by the injections of fentanyl (75 μg/kg, IV) in vehicle- or GSHee (100 μmol/kg, IV)-treated rats recorded over the 60 min period following injection of fentanyl. There were 9 rats in each group. **P* < 0.025, significant response. ^†^*P* < 0.025, GSHee *versus* vehicle. See text for the explanation of the abbreviations. Please note that the ANOVA plus multiple comparisons tests were done for each independent variable separately. As such, for Frequency of breathing for example, the ANOVA (and subsequent multiple comparisons testing) was constructed to analyze whether (a) the vehicle or GSHee responses were significant and whether the GSHee responses were different from the vehicle responses, and (b) whether the fentanyl responses were significant in the vehicle-treated or GSHee-treated rats and whether the fentanyl induced responses in the GSHee-treated rats were different from those in the vehicle-treated rats. Accordingly, for each variable there were 2 between-group comparisons and the modified P values was set at 0.05/2 = 0.025.

**Supplemental Table 3.**

Baseline ventilatory parameters and vehicle, glutathione (GSH) and fentanyl-induced responses

|  | **Frequency (breaths/min)** | |  | **Tidal Volume (ml)** | |  | **Minute Ventilation (ml/min)** | |
| --- | --- | --- | --- | --- | --- | --- | --- | --- |
| **Phase** | **Vehicle** | **GSH** |  | **Vehicle** | **GSH** |  | **Vehicle** | **GSH** |
| Pre (baseline) values | 102 ± 7 | 101 ± 5 |  | 2.48 ± 0.14 | 2.46 ± 0.15 |  | 254 ± 22 | 251 ± 19 |
| Peak drug response, % | +5 ± 4 | +41 ± 7*^,†^ |  | +4 ± 3 | +15 ± 4*^,†^ |  | +11 ± 7 | 61 ± 8*^,†^ |
| Total drug response, % | +1 ± 3 | +13 ± 3*^,†^ |  | +1 ± 3 | +2 ± 3 |  | +2 ± 4 | 15 ± 3*^,†^ |
| Fentanyl – peak decrease, % | -35 ± 6* | -38 ± 5* |  | -51 ± 7* | -49 ± 6* |  | -66 ± 9* | -64 ± 8* |
| Fentanyl – total decrease, % | -19 ± 3* | -14 ± 3* |  | -33 ± 4* | -31 ± 5* |  | -46 ± 7* | -40 ± 6* |

GSH, glutathione. The peak drug (vehicle or GSH at 100 μmol/kg, IV) and fentanyl (75 μg/kg, IV) responses are expressed as %change from Pre-values. The total changes elicited by drug (vehicle, GSH) or fentanyl represent the sum of the 15 individual %change from pre values. The data are presented as mean ± SEM. There were 9 rats in each group. *P < 0.05 comparisons, significant change from Pre-values. ^†^P < 0.05/3 comparisons, GSH *versus* Vehicle.

**Supplemental Figure 2**

**Supplemental Figure 2.** Effects of fentanyl (75 μg/kg, IV) on frequency of breathing (top panel), tidal volume (middle panel) and minute ventilation (bottom panel), in rats pretreated with vehicle (VEH; 1 ml/kg, IV) or glutathione (GSH, 100 μmol/kg, IV). The data are presented as mean ± SEM. There were 9 rats in each group. The stippled horizontal line denotes average resting values before injection of GSHee or vehicle.

**Supplemental Table 4.**

Changes in inspiratory and expiratory times and their ratio at three time of the study

|  |  | **Actual Values** | | |  | **%Change from Pre** | |
| --- | --- | --- | --- | --- | --- | --- | --- |
| **Parameter** |  | **Pre** | **+5 min** | **+30 min** |  | **+5 min** | **+30 min** |
| **Ti, sec** |  | 0.22 ± 0.01 | 0.35 ± 0.03* | 0.38 ± 0.03* |  | +61 ± 7* | +74 ± 8* |
| **Te, sec** |  | 0.32 ± 0.02 | 0.81 ± 0.09* | 0.31 ± 0.03 |  | +152 ± 16* | -4 ± 6 |
| **Ti/Te** |  | 0.68 ± 0.03 | 0.43 ± 0.04* | 1.23 ± 0.15* |  | -37 ± 5* | +81 ± 9* |

Ti, inspiratory time; Te, expiratory time; The data are presented as mean ± SEM. There were 9 rats in each group. *P < 0.05/2 comparisons, significant change for +5 min and/or +30 min values from Pre-values for each parameter. ^†^P < 0.05/3 comparisons, +30 min versus +5 min values for the 3 parameters.
